# Supplementary material for: The Various Packing Structures of Tb@C82 (I, II) Isomers in Their Cocrystals with Ni(OEP)
Source: Nanomaterials (Basel). 2023 Mar 9;13(6):994. doi: 10.3390/nano13060994 (PMC10054076; doi:10.3390/nano13060994)
Supplement: Supplementary file 1 [file nanomaterials-13-00994-s001.zip › nanomaterials-2246934-supplementary.pdf]

Article

The Various Packing Structures of Tb@C_{82} (I, II) Isomers in Their Cocrystals with Ni(OEP)

Wei Dong [1], Qin Zhou [1], Wangqiang Shen [2], Le Yang [3], Peng Jin [3], Xing Lu [2,*] and Yongfu Lian [1,*]

[1] Key Laboratory of Functional Inorganic Material Chemistry, Ministry of Education, School of Chemistry and Materials Science, Heilongjiang University, Harbin 150080, China; dongweivvv@163.com (W.D.); zhouqin@hlju.edu.cn (Q.Z.)
[2] State Key Laboratory of Materials Processing and Die & Mould Technology, School of Materials Science and Engineering, Huazhong University of Science and Technology, 1037 Luoyu Road, Wuhan 430074, China; shenwq@hfut.edu.cn
[3] School of Materials Science and Engineering, Hebei University of Technology, Tianjin 300130, China; yangle08092023@163.com (L.Y.); china.peng.jin@gmail.com (P.J.)
* Correspondence: lux@hust.edu.cn (X.L.); chyflian@hlju.edu.cn (Y.L.)

Synthesis of Tb@C_{82} isomers

Carbon soot containing Tb-EMFs was synthesized using a direct current arc discharge method. Briefly, a core-drilled graphite rod filled with graphite/Tb_4O_7 (molar ratio: Tb/C=1:15) was installed in the anode position of the electric arc furnace, and the solid graphite rod is installed in the cathode. Before the preparation of EMFs, it is necessary to short-connect the negative and the positive poles, turn on the current (100 A), and perform online preheating for 30 minutes. After that, the air chamber was evacuated until the vacuum degree was below 5 Pa, and then a certain volume of inert gas (helium, 400 mbar) was injected, and current intensity was set 100 A. Finally, the two electrodes were discharged and arced, and the distance between the two electrodes was maintained between 3 and 5 mm. Normally, it takes about 15 minutes for a hollow graphite rod to be completely consumed. After the graphite rod was consumed, the DC arc machine was turned off, cooled for about 30 min until the gas chamber was completely cooled, and soot was collected.

Differential pulse voltammetry (DPV) characterization of Tb@C_{82} isomers

A 25 mL detection cell was selected for the test, the working, counter and reference electrodes are a platinum disc, a platinum plate and an Ag wire, respectively. The toluene solution of Tb@C_{82} was evaporated by a rotary evaporator, and the solid product was dissolved in 3 mL of 0.05 M o-DCB solution of n-Bu_4NPF_6 with the assistance of ultrasound wave. After filtration with a 0.2 μm PTFE membrane, the filtrate was taken into the detection cell, and then Ar was passed to exclude O_2 and other impurities. The test of DPV was conducted with a sweep speed of 20 mV/s and in the window of -2.5 ~ 2.5 V.

Shown in Figure S4 are the DPV curves of Tb@C_{82} isomers. It can be seen that Tb@C_{82} (I) exhibits two pairs of reversible oxidative and five pairs of reversible reductive peaks, and Tb@C_{82} (II) shows two pairs of reversible oxidative and five pairs of reversible reductive ones, consistent with the results of CV characterization.

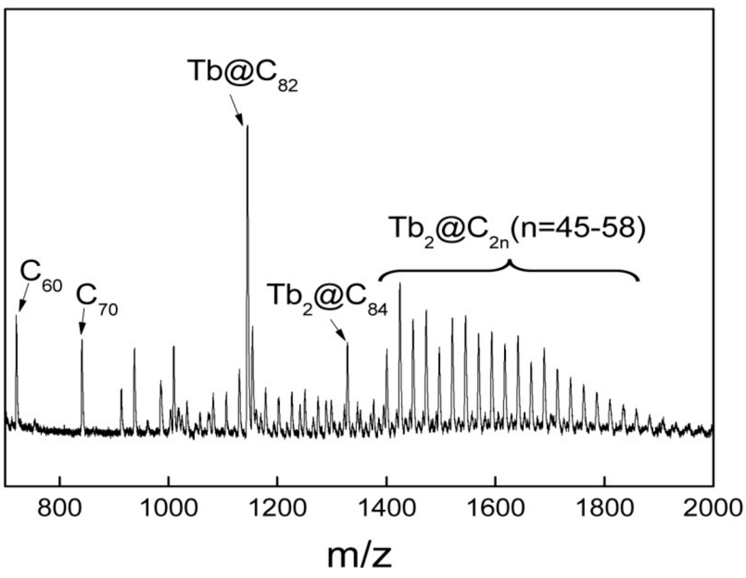

Figure S1. MALDI-TOF mass spectrum of the TCB extract.

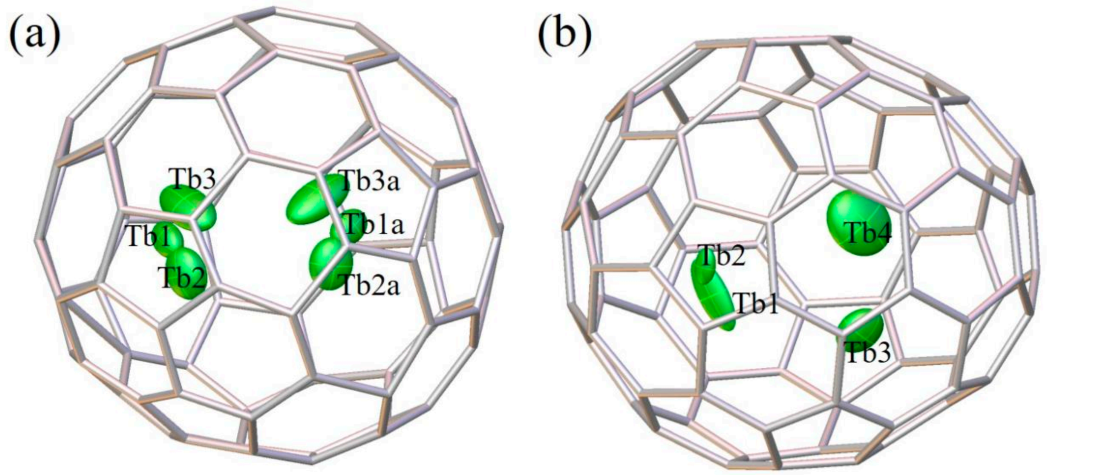

Figure S2. Positions of the disordered Tb atom in (**a**) Tb@C_{2v}(9)-C_{82} and (**b**)Tb@C_s(6)-C_{82} with respect to C_{82} cage orientation, respectively.

Table S1. Relative energies (kcal/mol) of Tb@C_s(6)-C_{82} and Tb@C_{2v}(9)-C_{82} with different spin multiplicities (*M*).

M	**6**	**8**
Tb@C_s(6)-C_{82}	**12.3**	50.1
Tb@C_{2v}(9)-C_{82}	33.1	**0.0**

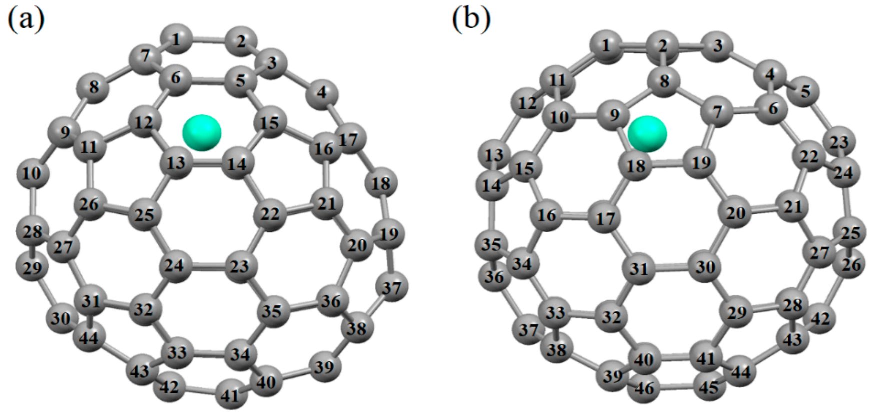

Figure S3. Numbering scheme of the unequivalent cage carbon atoms in (**a**) Tb@C_s(6)-C_{82} and (**b**) Tb@C_{2v}(9)-C_{82}. Cage carbon atoms at the equivalent sites are omitted for clarity.

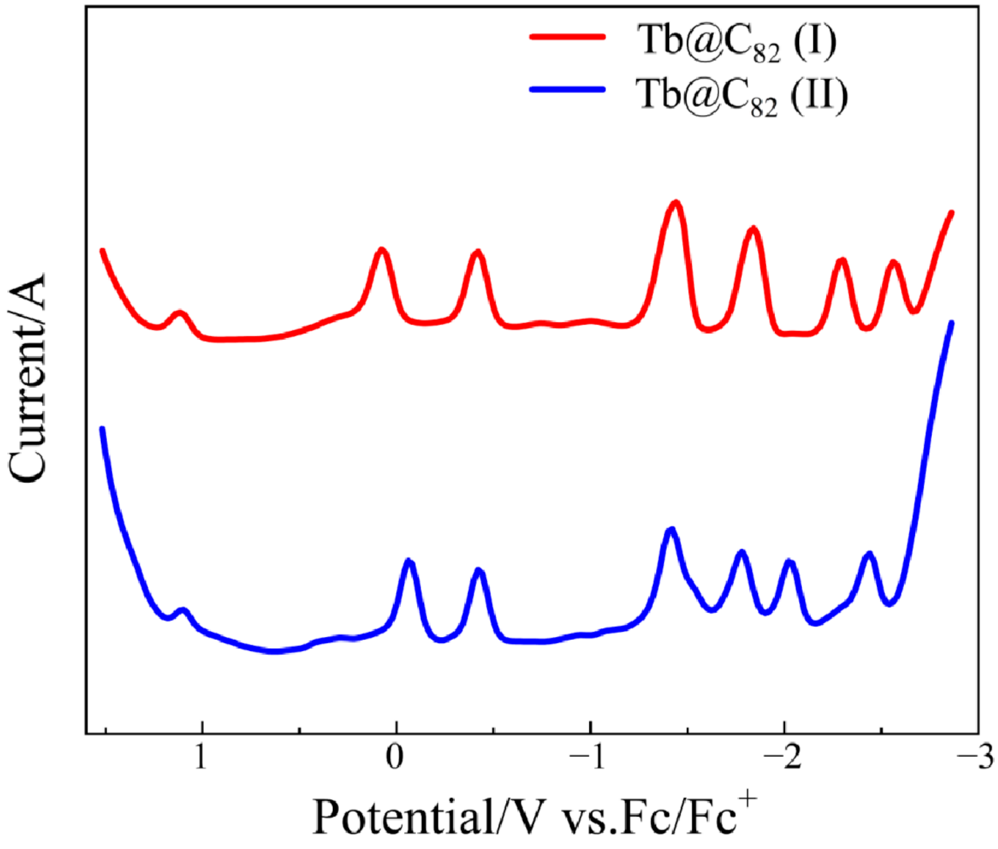

Figure S4. DPV curves of Tb@C_{82} isomers. Conditions: working electrode, Pt wire; counter electrode, Pt wire; reference electrode, Ag /AgCl; supporting electrolyte, 0.05 M n-Bu_4NPF_6 in 1,2-dichlorobenzene; scan rate, 20 mV/s; pulse amplitude = 50 mV.

Table S2. The crystallographic data of [Tb@C_{2v}(9)-C_{82}]·[Ni(OEP)]·2(C_6H_6) and 2[Tb@*Cs*(6)-C_{82}]·2[Ni(OEP)]·1.5(C_6H_6)·(CS_2)

Compound	[Tb@C_{2v}(9)-C_{82}]·[Ni(OEP)]·2(C_6H_6)·	2[Tb@*Cs*(6)-C_{82}]·2[Ni(OEP)]·1.5(C_6H_6)·2(CS_2)
**T, K**	100	100
**λ, Å**	0.71069	0.8856
**color/habit**	black / block	black / block
**Empirical formula**	$C_{130}H_{56}N_4NiTb$	$C_{247}H_{97}N_8Ni_2S_4Tb_2$
**crystal system**	monoclinic	monoclinic
**space group**	*C*2/*m*	*P*2₁/C
**a, Å**	25.315	17.9531
**b, Å**	15.197	17.0851
**c, Å**	19.892	26.4719
**α, deg**	90.000	90.000
**β, deg**	94.891	108.1984
**γ, deg**	90.000	90.000
**V, Å^3**	7624.9	7713.6
**Z**	2	2
**ρ, g/cm^3**	1.648	1.661
**μ, mm^{-1}**	0.993	2.264
**Data/restraints/parameters**	7044/930/780	16390/3259/1780
**R1 [reflections with I>2σ(I)]**	0.1018(5952)	0.0522(12308)
**wR2 (all data)**	0.2794(7044)	0.1319(16390)
